# A Review of Complement Activation in SLE

**DOI:** 10.1007/s11926-021-00984-1

**Published:** 2021-02-10

**Authors:** Arthur Weinstein, Roberta V. Alexander, Debra J. Zack

**Affiliations:** 1 Loma Linda University, Loma Linda, CA USA; 2 Georgetown University, Washington, DC USA; 3 Exagen Inc., Vista, CA USA

**Keywords:** Systemic lupus erythematosus, Complement, Complement activation measurement, Disease activity, Lupus nephritis

## Abstract

**Purpose of Review:**

Complement activation is a key event in the pathogenesis of tissue inflammation and injury in systemic lupus erythematosus (SLE). This review is aimed at comparing the usefulness of measurement of complement proteins in serum/plasma (C3, C4) to complement activation (split) products in plasma and on circulating blood cells for SLE diagnosis, disease monitoring, and prognosis.

**Recent Findings:**

Complement split products, C3dg, iC3b, and C4d, are elevated in SLE, and C3dg/C3 and iC3b/C3 ratios correlate with active SLE. C4d also is higher in patients with lupus nephritis. An elevated level of the alternative pathway split product, Bb, in early lupus pregnancy is a predictor of adverse outcomes in SLE patients with antiphospholipid antibodies. Elevated levels of cell-bound complement activation products (CB-CAPs), namely, B cell-bound C4d (BC4d) and erythrocyte-bound C4d (EC4d), within a multiparameter assay panel, may predict transition to SLE more than other lupus biomarkers. EC4d better correlates with lupus disease activity than low plasma complement levels. Elevated platelet-bound C4d (PC4d) correlates with thrombosis in SLE. Both EC4d and PC4d are increased in primary and secondary anti-phospholipid syndrome, and anti-beta2glycoproteinI antibodies may directly activate the complement system.

**Summary:**

Abnormal levels of plasma complement split products and CB-CAPs support complement activation as an important pathogenetic mechanism in SLE and the antiphospholipid syndromes. These tests show promise for the diagnosis of SLE and monitoring of disease activity.

## Introduction

**Fig. 1:**
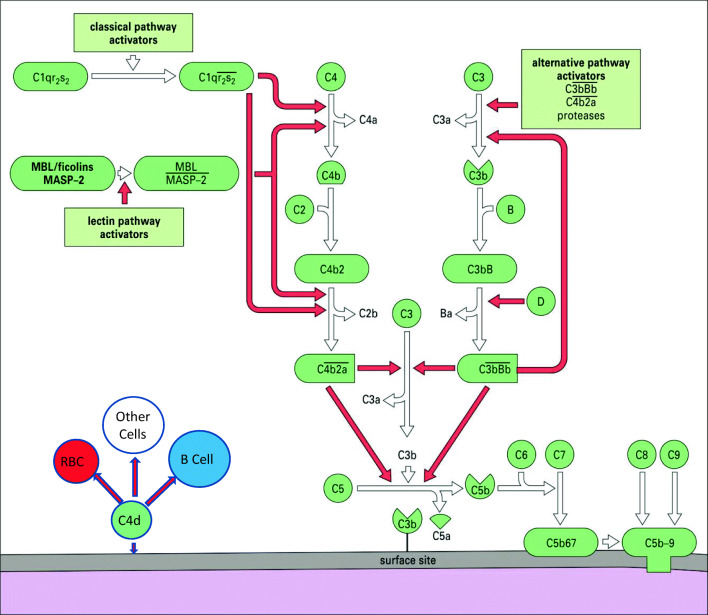
Pathways of complement activation. Classical pathway activators include surface-bound IgG and IgM and circulating immune complexes. MBL mannose-binding lectin, MASP MBL-associated serine proteinases. Red arrows show feedback (amplification) loops. Modified from Morgan BP: Complement. In: Male D, Brostoff J, Roth DB, Roitt IM, editors. Immunology. 8th edition, Elsevier, 2012

It has been 120 years since the descriptions of the bactericidal activity in normal serum and the elucidation of the role of complement in normal human physiology and disease [1]. It is now known that the complement system consists of more than 30 plasma proteins and cell surface receptors involved in the activation and regulation of its lytic functions [1, 2]. There are three pathways of complement activation (Fig. 1), all of which may be involved in systemic lupus erythematosus (SLE) inflammation and tissue damage, with the classical pathway, activated by antigen-antibody complexes, being the most important. The complement system has many biological functions in addition to bacteriolysis which may be important in SLE pathophysiology including promotion of inflammatory processes, clearance of immune complexes, and clearance of cellular and apoptotic debris [2]. Involvement of the complement system in SLE has been described by many investigators over the past 70 years with low levels of complement proteins (C3, C4) and hemolytic activity having potential use as diagnostic markers for SLE and for monitoring disease activity [3–5]. However, limitations in using complement levels in SLE have been well documented [6, 7]. Methods to detect complement activation in SLE and other conditions are shown in Table 1 (adapted from [7]).

**Table 1:** Detection of in vivo complement activation

Measurement	Example
Individual complement plasma proteins	C3, C4, factor B, and other pathway proteins decreased
Total hemolytic complement (snap frozen plasma)	CH_50_ decreased
Individual complement protein metabolism	C3 hypercatabolism
Deposit of complement proteins in tissues	Detection of C3, C4, C1q, C4d, and membrane attack complex (MAC, C5b-9) on glomeruli and skin basement membranes
Plasma activation (split) products	C3dg, iC3b, C4d, MASP-2, C5a, Bb, MAC, and others increased
Cell-bound complement activation products (CB-CAPs)	B cell C4d (BC4d), erythrocyte C4d (EC4d), and platelet-bound C4d (PC4d), among others, increased

This article will provide the current state of the art in the detection of complement activation in SLE since its last review in this journal [7]. We will review the use of traditional complement proteins, C3 and C4, and compare them to complement activation (split) products in plasma and on cell surfaces in relation to their application to SLE diagnosis and monitoring of disease activity.

## Plasma/Serum Complement Proteins as Markers of Complement Activation in the Diagnosis of SLE

SLE is recognized as a disease where autoantibodies develop and fix to self-antigen resulting in complement activation and in turn leading to inflammation and tissue damage. Plasma complement levels can be influenced by a variety of factors: complement increases during the acute phase response, individual variability in complement gene copy number and expression, and variability in protein synthesis and catabolism. All these can influence plasma/serum complement levels [7]. Because of these factors, low complement levels perform poorly as diagnostic markers for SLE.

The SLE classification criteria modified over the years reflect the difficulty of utilizing serum complement levels. These criteria were developed for inclusion of SLE patients in research studies, and because of the poor sensitivity of low complement levels in lupus, the original criteria developed by the American College of Rheumatology did not have hypocomplementemia as a criterion [8]. The new classification criteria developed by the ACR and the European League against Rheumatism (EULAR) do include low plasma complement (C3, C4 CH_50_), but partly because of expert opinion rather than statistical modeling [9].

In a European multinational inception cohort of 200 newly diagnosed (within two years) lupus patients, many of whom had active disease, low C3 and/or C4 levels were observed at baseline in 54% [10]. Low complement levels are found even more infrequently in very early and milder disease. In a recent study of patients with probable lupus who fulfilled only three ACR criteria, only 36% had low C3 and/or C4 historically even though 30% transitioned to SLE at follow-up [11••].

## Complement Levels in the Monitoring of SLE Disease Activity and Lupus Nephritis

Biomarkers of disease activity and flare must be studied longitudinally rather than cross-sectionally in lupus populations. With the interaction of complement proteins, receptors, inhibitors, and autoantibodies, this can be a complicated investigation. This is made more challenging by the variable natural history of SLE [12].

Low complement levels have proven disappointing as disease activity markers in general SLE because of both persistently low or normal levels, independent of disease activity, and insensitivity at predicting flares [6, 13, 14•]. Although the older studies suggest that low complement levels may reliably correlate with flares of lupus nephritis, this was not confirmed in a study of 71 lupus nephritis patients followed prospectively for about three years [15]. In the documented 70 flares, levels of C3 or C4 did not decrease in about one third of the patients giving a sensitivity of C3 and C4 for flare of 70% and 49%, respectively. In a multivariate regression analysis, they showed that significant decreases in C4 did occur about two months before the renal flare in some patients. Also, a decrease in C3 that occurred at the time of flare was influenced by genotypic variation in factor H, a regulator of C3 convertase in the alternative pathway. These data, which remain to be confirmed, indicate the complex involvement of complement activation in lupus nephritis and the limitations in using complement levels for disease monitoring. Another prospective study of 228 patients with lupus nephritis over six years showed that an increase in autoantibodies to C1q outperformed low complement levels in the correlation with renal flares, especially in proliferative lupus nephritis, with 80.5% sensitivity and 71% specificity [16]. However, 20% of patients had a renal flare with normal anti-C1q levels and 30% in renal remission had high anti-C1q levels. Another longitudinal study demonstrated that a rising titer of both anti-C1q and anti-ds DNA together could predict a renal flare in some patients by many months [17].

## Complement Activation (Split) Products in the SLE Disease Monitoring and in Lupus Nephritis

### Analyses of Plasma Split Products

The above-described limitations of the complement proteins C3 and C4 as biomarkers for the diagnosis and monitoring of SLE disease activity have prompted the development of assays to measure the proteolytic fragments of complement proteins. As these fragments are formed upon activation of the complement cascade, complement split products reflect complement activation more accurately than the levels of the individual intact proteins.

Assays for several complement split products—either soluble or cell-bound—have been developed to evaluate whether these fragments can serve as biomarkers of SLE either to aid in the diagnosis of the disease or to monitor disease activity or as prognostic markers for flares of nephritis. There are technical issues related to detection of these split products in plasma. Many have a very short half-life. Also, some complement activation can occur in vitro at room temperature, even when collected in EDTA plasma tubes. Therefore, the split products must be measured within a short time on fresh plasma or the sample must be snap frozen until studied. These challenges have made these tests impractical for general clinical use outside of research laboratories [7]. However, the information gleaned from the recent studies described below adds to the accumulating evidence that complement activation in vivo in SLE is better detected by the presence of split products than by low complement protein levels.

Complement fragments derived from the classical pathway (C4d) or from the convergence of the three pathways of complement activation (C3dg, iC3b) hold promise as biomarkers of SLE.

C3dg has a half-life of 4 h and can be measured in EDTA plasma frozen within a few hours of collection. Samples appear stable at −80 °C for a year, and values are not significantly affected by up to four freeze-thaw cycles. The assay for C3dg requires a step for the precipitation of larger C3 fragments. The supernatant can then be tested with the time-resolved immunofluorimetric assay or ELISA [18•]. Because the formation of split products in SLE happens in parallel with the degradation and synthesis of C3 during complement activation, the ratio C3dg/C3 has also been evaluated. The performance characteristics of C3dg and the C3dg/C3 ratio have been compared with C3 levels in SLE and normal healthy subjects. Areas under the curve (AUC) of the receiver operating characteristics (ROC) are high at 0.96 and 0.89, respectively, and much better than those of C3 levels; however, comparison of SLE versus other rheumatic diseases has not been performed [18•].

iC3b has a half-life of 90 min. Samples of blood, plasma, or serum are placed within 30 min of venipuncture in a buffer that prevents spontaneous complement activation and are frozen immediately. The levels of iC3b and the iC3b/C3 ratio are higher in SLE patients than in normal healthy subjects, and the SLE patients with active disease have iC3b and iC3b/C3 ratio higher than patients with inactive disease. The AUC of the ROC curve of iC3b/C3 is higher than that of iC3b in discriminating active and inactive SLE and flaring vs. nonflaring patients, and it outperformed C3 and C4 levels. In fact, the majority of patients with active SLE had high iC3b and iC3b/C3 ratio whereas only 37% had low C3 levels [19•]. The quantitative lateral flow assay used in this study mitigates some of the handling issues with split product determination which can lead to in vitro complement activation [20].

C4d is a promising marker of disease activity in SLE as the delta change in plasma C4d values between low and high disease activities in a certain patient was higher for C4d than for C3 and C4 and the AUC of the ROC curve for high disease activity was higher for C4d than for C3 and C4 [21•]. In addition, C4d was higher in patients with nephritis than in patients without renal involvement. Of note, although the odds ratios of C4d were higher than those of C3 and C4, they were lower than those of dsDNA. The combination of anti-dsDNA and C4d was significantly associated with nephritis. In addition, data in a small number of subjects also indicate that C4d may be able to predict a lupus nephritis flare in patients who had already an episode of nephritis and, thus, are at increased risk. This study was conducted with plasma samples isolated from blood within 1 h of venipuncture and frozen within 2 h. The ELISA was performed with an anti-C4d antibody that recognizes a neoepitope formed after C4b cleavage to C4d. The same group also analyzed the C4d/C4 ratio in a different patient population and found that both C4d and the C4d/C4 ratio were higher in SLE patients—and especially in lupus nephritis—than in healthy controls [22••]. The AUC of the ROC curve of the C4d/C4 ratio for lupus nephritis was 0.76, higher than that of C4d and C4 alone (both 0.71). In addition, odds ratio analysis showed that high C4d, low C4, and low C3 were associated significantly with nephritis with the C4d/C4 ratio having the highest relative odds. Importantly, C4d and the C4d/C4 ratio decreased in lupus nephritis patients who responded to therapy—while they did not change in nonresponders—and are associated with histopathological changes. The association with both clinical and histopathological responses in lupus nephritis suggests that the determination of C4d in plasma may at least partially replace invasive biopsies to evaluate active nephritis.

Limited data exists on soluble complement fragments derived from the lectin pathway. Some proteins of the lectin pathway are lower in SLE patients than in controls, while others have the opposite trend. In addition, some display a negative correlation with disease activity and a positive correlation with C3, indicating that consumption of some of the proteins of the lectin pathway occurs in SLE. In some studies, significant differences in concentrations between patients with SLE and controls have been observed, and in one study, there was a correlation of MASP-2 levels with SLE disease activity [23]. However, the wide range of the concentrations of these proteins in patients and controls makes their use as biomarkers of SLE uncertain at this time. Genetic deficiencies of mannose-binding lectin (MBL) have been associated with SLE [24].

### Cell-Bound Complement Activation Products (CB-CAPs) as Markers in SLE

C4d as well as C3d is not only present in solution but also bound to the membrane of blood cells, such as erythrocytes, reticulocytes, B and T lymphocytes, and platelets. These complement fragments which are covalently bound to cell membranes are collectively called cell-bound complement activation products (CB-CAPs) [7]. They can be measured in EDTA-anticoagulated blood by flow cytometry, and their stability allows for samples to be shipped refrigerated to a central laboratory with overnight delivery. Although flow cytometry is labor intensive, sample processing in the clinic is minimal and does not require centrifugation or storage and transportation at low temperatures.

Extensive research has demonstrated the value of CB-CAPs to aid in the diagnosis of SLE as well as to monitor disease activity, and a recent review on CB-CAPs in SLE has been published [25•]. In essence, CB-CAPs are more sensitive than plasma/serum levels of C3 and C4 for the diagnosis of SLE. A more recent study showed that CB-CAPs (BC4d, EC4d) are more prevalent than low complement levels in patients with probable SLE [11••] and when used in a multiparameter assay panel are better than other biomarkers at predicting transition to SLE.

We will review here recent data on C4d bound to erythrocytes (EC4d) and platelets (PC4d).

An analysis of three independent SLE populations showed that EC4d is associated with disease activity measured with the SELENA-SLEDAI or with the physician global assessment. Association is increased when EC4d is combined with low complement C3/C4, indicating that both biomarkers have value in monitoring disease activity in SLE. Interestingly, in the majority of patients, serum complement levels did not change over time and were chronically low or chronically normal. In this subset, EC4d remained associated with disease activity, suggesting that EC4d is superior to C3/C4 and can give information on disease activity in patients whose C3/C4 does not reach abnormally low levels during high disease activity [14•].

Because of the increased risk of thrombosis in SLE, recent studies have evaluated whether CB-CAPs are associated with history of thrombotic events. EC4d has weak association, and BC4d is not associated with thrombosis. PC4d, however, has strong association with history of thrombosis (odds ratio = 8.4). The association is particularly strong with venous thrombosis (odds ratio = 19.2) and weaker with arterial thrombosis (odds ratio = 4.0) [26••]. Multivariate logistic regression revealed that PC4d, low C3, and lupus anticoagulant are all significantly and independently associated with thrombosis, indicating additive utility, especially for venous thrombosis.

Association of PC4d with history of thrombosis was recently found also by another group that analyzed PC4d by flow cytometry utilizing frozen platelet-rich plasma [27•] instead of fresh blood. Comparison of PC4d in patients with SLE to controls in the general population showed that PC4d was associated with venous thromboembolism and ischemic stroke, but not with ischemic heart disease or subclinical atherosclerosis. Interestingly, this study found not only a negative correlation between PC4d and C3 or C4 but also a positive correlation between PC4d and C3dg and, to a lesser extent, with soluble C5a-9 [27•]. This suggests that both soluble and cell-bound complement split products may be useful biomarkers in SLE and may be associated with thrombosis. Complement-mediated thrombosis has been found in many complementopathies, even though the mechanisms remain unclear [28].

The ability of PC4d to predict the occurrence of cardiovascular events has yet to be demonstrated. In fact, prospective studies on thrombosis in SLE are particularly challenging owing to the relatively low number of events over a long period of time.

## Complement Activation with Other Lupus Features

### Antiphospholipid Syndrome (APS)

The risk of thrombosis in lupus depends on abnormalities such as known antiphospholipid antibodies (aPL), lupus anticoagulant, and nephrotic syndrome but as described above also markers of complement activation—low C3 and PC4d [26••]. Complement activation with hypocomplementemia is known to be associated with the primary APS as well as APS in SLE [29].

The link between thrombosis and complement activation was explored in two recent studies. Lonati and colleagues [30•] showed that EC4d and PC4d were increased in primary and secondary APS and correlated with serum anti-beta2glycoprotein1 and anticardiolipin antibodies. They also showed that a monoclonal antibody against the D1 domain of anti-beta2glycoprotein1 can bind in vitro to activated, but not resting, platelets leading to complement activation and deposition of C4d on the platelet surface. Although this study showed that C4d was deposited on a small percentage of platelets upon the addition of the autoantibody, it is tempting to speculate that immuno complexes containing anti-phospholipid and possibly other autoantibodies activate the complement cascade and lead to increased PC4d levels in patients whose platelets are not in a resting state. Thus, PC4d may be a marker of both complement and platelet activation in SLE. Another recent study provided evidence for complement activation in thrombotic APS as well as catastrophic APS (CAPS). It showed that anti-beta2glycoproteinI antibodies activated complement directly and contributed to thrombosis whereas mutations in complement regulatory genes led to uncontrolled complement activation in CAPS [31••].

Patients with SLE and/or APS are known to have an increased risk of adverse pregnancy outcomes (APOs) of fetal death, neonatal death, preterm delivery, and small gestational age infants. In a study of nearly 500 pregnant patients with SLE and/or antiphospholipid antibodies, patients with APOs showed increased levels of an alternative pathway split product, Bb, as well as the terminal pathway component MAC (sC5b-9) early in pregnancy (12–15 weeks) [32••]. Alternative complement pathway activation is strongly implicated as a contributor to abnormal placental development and APOs.

### Thrombotic Microangiopathy (TMA)

Thrombotic microangiopathy (TMA) is an endothelial injury that occurs in 1–4% of lupus nephritis kidneys and is associated with severe morbidity and high mortality. Over half of lupus patients with TMA have complement regulatory protein mutations that have been associated with atypical hemolytic uremic syndrome (aHUS—microangiopathic hemolytic anemia, thrombocytopenia, and renal impairment). This has also been found in TMA associated with lupus nephritis [33]. Given the importance of complement fixation in lupus nephritis kidneys, a meta-analysis of complement inhibiting therapy in lupus nephritis with TMA was carried out. Fourteen studies were identified, with 30 patients included. TMA was determined either histologically or by aHUS diagnosis. All were treated with eculizumab, an inhibitor of C5 activation. A majority (28/30) of the patients had resolution of symptoms and recovery of renal function or were able to be discharged from the hospital. This underscores the interest in the development of complement inhibitory medications and the need for markers to determine which patients will benefit the most from them [34•].

## Complement Activation and Cell Function

Activation of complement may affect the cells to which the activated products bind which might augment lupus-related dysfunction and inflammation. C4d deposited on erythrocytes in trauma patients can decrease red cell deformability which could affect oxygen delivery to tissues [35]. C3d deposited on T cells in SLE results in greater production of cytokines—interferon gamma and interleukins 4 and 17 [36]. A recent review highlighted the role of the intracellular complement system (complosome) and its activation on T cell metabolism [37].

## Conclusion

There is strong evidence that activation of the classical complement pathway by immune complexes in SLE is the terminal event leading to tissue damage in many organs. Despite this, low levels of C4 and C3 have limited diagnostic utility in SLE and poorly reflect disease activity. Newer studies measuring plasma complement split products and cell-bound activation products have suggested that elevated levels of these fragments are more useful diagnostic markers and more tightly correlate with SLE disease activity. Furthermore, there is growing evidence for the link between complement activation and thrombosis in SLE and in APS. With complement therapeutics gaining increasing use, it will be important to select those SLE patients who clearly have demonstrable evidence of complement activation and for whom this therapy may be most efficacious. Therefore, newer more reliable tests, as described in this review, may prove to be clinically useful.
